# Successful treatment of a pure red-cell aplasia patient with γδT cells and clonal TCR gene rearrangement: A case report

**DOI:** 10.3389/fimmu.2022.1103448

**Published:** 2023-01-16

**Authors:** Xian Li, Xinyi Zhu, Xiaohong Zhang, Weiqin Wang

**Affiliations:** ^1^ Department of Hematology, the Second Affiliated Hospital of Zhejiang University School of Medicine, Hangzhou, China; ^2^ Faculty of Medicine, The University of Queensland, Brisbane, QLD, Australia

**Keywords:** pure red-cell aplasia, flow cytometry, γδT cell, T-cell receptor gene rearrangement, case report

## Abstract

Pure red-cell aplasia (PRCA) is a syndrome associated with reduced erythroid precursors. This report presents the case of an elderly PRCA patient with significantly proliferated γδT cells and clonal T-cell receptor (TCR) gene rearrangement. The cause of this patient’s PRCA was confirmed to be an autoimmune disorder rather than malignancy on the basis of flow cytometry, TCR gene rearrangement, and positron emission tomography/computed tomography (PET/CT) findings. Moreover, the γδT cell group identified in this case was captured for the first time under the microscope; this CD4+/CD8− (extremely high CD4/CD8 ratio) population is rare in PRCA patients. Our patient with a monoclonal and polyclonal hybrid of TCR gene rearrangement was sensitive to cyclosporin A (CsA), despite previous reports suggesting that patients with TCR clonal rearrangement may respond poorly to this drug. Overall, this case presents valuable clinical findings for the future diagnosis and management of PRCA caused by autoimmune conditions and further research on γδT cells’ autoimmune pathophysiology and gene rearrangement.

## Introduction

Pure red-cell aplasia (PRCA) is a rare disorder and one cause of severe normochromic and normocytic anemia. PRCA can be congenital or acquired. Acquired PRCA can result from autoimmune disorders with or without lymphoproliferative disorders, exposure to certain drugs, and infections, particularly human parvovirus B19 (1). Lymphoproliferative disorders most observed and reported to be associated with PRCA are lymphoid malignancies of either B- or T-cell type (2, 3). γδT cells were the primary proliferative cell type found in the present case and are an innate immune subset of T cells existing in small numbers (normally <5% of all T cells), mainly found in the mucosa, skin, and spleen (4). The clonal expansion of γδT cells is commonly reported in patients with lymphomas in these restricted sites. T-cell receptor (TCR) gene rearrangement, observed in some PRCA patients, may play a role in PRCA pathogenesis. PRCA patients with TCR clonal rearrangement with TCRδ or/and TCRγ may respond poorly to cyclosporin A (CsA) (5).

The present report describes the case of a 77-year-old PRCA patient who presented with fatigue, dizziness, syncope, and skin itchiness. A significant proliferation of multiclonal γδT cells was identified in the patient’s bone marrow and peripheral blood. PRCA cases with CD4+/CD8− (extremely high CD4/CD8 ratio) γδT cells were rare in previous reports (1–3). TCR gene rearrangement revealed monoclonal patterns in several different TCR gene segments, consistent with its rarity. However, the patient was sensitive to CsA, indicating a benign lymphocyte proliferation.

## Case report

A 77-year-old male patient visited the emergency department of our hospital on 11 September 2020 due to recurrent syncopal episodes. At the time of review, he was afebrile and showed no signs of cardiovascular diseases, such as abnormal heart sounds. Other physical examinations showed pallor with no bleeding or bruises, lymphadenopathy, hepatosplenomegaly, or jaundice. The patient’s hemoglobin level was 37 g/L (normal range, 131–172), red cell count was 0.9 × 10^12^/L (normal range, 4.09–5.74), and reticulocyte fraction was 0.34% (normal range, 0.5–1.5). The white cell count was 3.3 × 10^9^/L (normal range, 4–10), and the platelet count was 181 × 10^9^/L (normal range, 100–300). The patient was immediately transfused until his symptoms resolved. When questioned about his history, the patient reported 6 months of recurrent fatigue, dizziness, syncope, and skin itchiness. Anemia was first noticed in him 1 year prior with a hemoglobin level of 99 g/L and a red cell count of 2.58 × 10^12^/L, but he did not report any dizziness or syncope at that time, and no treatment was provided.

The patient had multiple presentations in the subsequent 3 months with recurrent fatigue, dizziness, and syncope. He continued to show low hemoglobin levels (lowest at 25 g/L). Transfusion was ordered as immediate management, and the causes of his anemia were screened. The patient tested negative for human parvovirus B19, cytomegalovirus, human immunodeficiency viruses, hepatitis C virus, hepatitis B virus, and Epstein–Barr virus. His vitamin B12, folate, and iron levels were normal, and he was negative for autoantibodies (including the anti-erythropoietin antibody).

Bone marrow aspiration was then ordered. The bone marrow aspirate smear showed an absence of erythroblasts with normal distribution and differentiation of other hematopoietic lineages. PRCA was thus diagnosed. T-cell lymphoproliferative disorders were screened due to the negative results in the aforementioned investigations. T-cell receptor (TCR) gene rearrangement testing (fragment analysis by capillary electrophoresis) of the bone marrow samples and flow cytometry analyses of the peripheral blood and bone marrow were conducted.

Flow cytometry revealed the proliferation of γδT cells in the peripheral blood and bone marrow, accounting for 14.075% and 11.162% of all nucleated cells, respectively (**Supplementary Figures S1**, **S2**). The immunophenotype of these γδT cells was CD5−, CD7+, CD16+, CD56+/−, CD94+, CD335−, and CD337−. The CD4/CD8 ratio was 13.075%. Monoclonal TCR gene rearrangement was detected in TCRβ, TCRδ, and TCRγ, while multiclonal patterns were also detected in TCRβ and TCRγ (**Supplementary Figure S3**).

**Figure 1:**
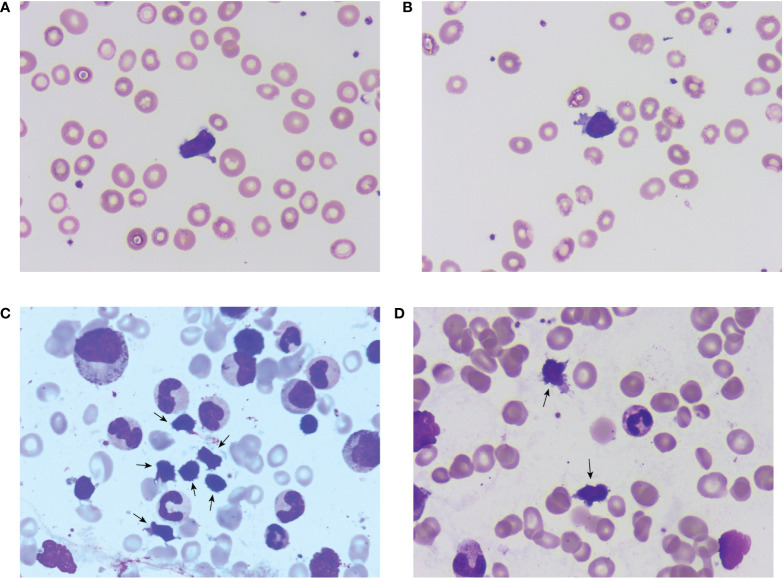
Microscopic morphology of the clonal γδT cell subtype. **(A, B)** show single cells found in a peripheral blood smear and show clear morphological characteristics; **(C, D)** are clustered cells found in the bone marrow aspirate smear with clear cytoplasmic protrusions.

Consistent with the aforementioned flow cytometry results and TCR rearrangement, a characteristic population of lymphoid cells was found in the peripheral blood and bone marrow smears (**Figures 1A–D**). These cells were not prominent and thus easily overlooked in the initial microscopic examination. A further thorough examination revealed that these cells were commonly clustered in distribution and shared a set of morphological features: small-sized lymphoid cells with a high nuclear/cytoplasmic ratio, oval or irregular nuclei, no visible nucleoli, slightly basophilic cytoplasm, and, most importantly, a small number of azurophilic granules and numerous evident protrusions. These cells resembled the shape of cocklebur seeds and were captured for the first time under a microscope in a real clinical case.

It was suspected that the proliferation of γδT cells caused the patient’s PRCA. However, the cause of this proliferation needed to be identified for proper management. A whole-body PET/CT was ordered, and the result showed no significant sign of lesions. Intestinal T-cell lymphoma, hepatosplenic T-cell lymphoma, primary cutaneous γδT cell lymphoma, and T-cell large granular lymphocytic leukemia were inconsistent with the cells’ normal immunophenotype, the multiclonal nature of the TCR gene rearrangement, and the patient’s clinical presentations; thus, these diseases were excluded. The observed proliferation of γδT cells was thus confirmed to be the primary proliferative disease and was categorized as an autoimmune disorder.

**Figure 2:**
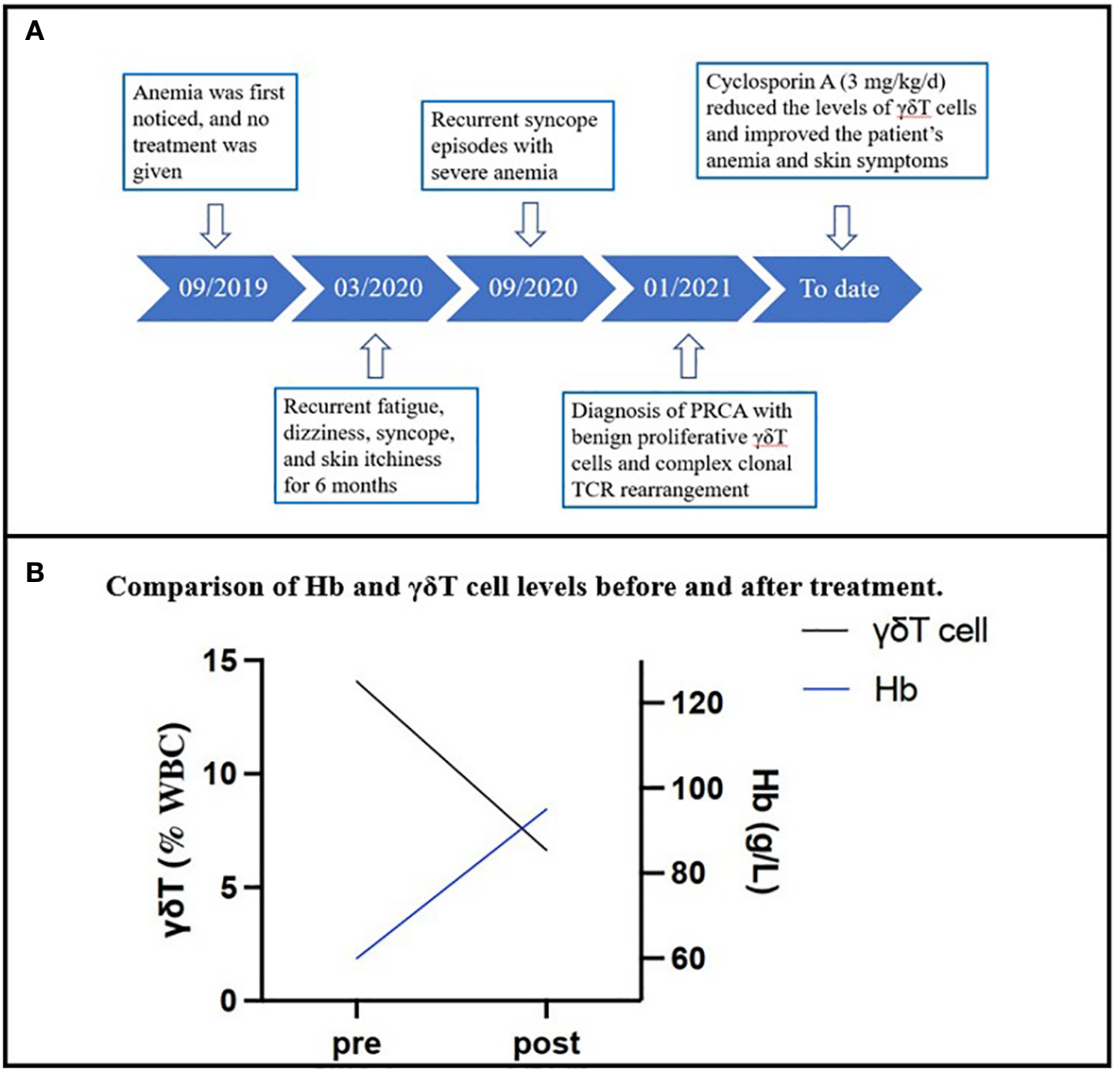
**(A)** The timeline of the case. **(B)** The change in the patient’s hemoglobin levels and γδT cell percentage in the total T-cell component in the peripheral blood sample over 10 months.

The patient’s renal function was checked, and he was then administered CsA (3 mg/kg/d). One month later, the follow-up blood test revealed that the patient’s hemoglobin level was 60 g/L, his red cell count was 1.52 × 10^12^/L, and his reticulocyte fraction was 2.9%. The white blood cell count was 4.8 × 10^9^/L, and the platelet count was 329 × 10^9^/L. His skin itchiness, which previously disturbed his sleep, resolved immediately after treatment. The patient’s peripheral blood hemoglobin level increased from 37 to 95 g/L after 10 months of treatment, and his γδT cell percentage decreased from 14.075% to 6.637% (**Figure 2B**). To date, follow-up blood tests have demonstrated that his hemoglobin levels are stabilized between 95 and 99 g/L, similar to his levels before the onset of symptoms. Although his hemoglobin level remains slightly below the lower normal range of a male (120 g/L) and his γδT cell percentage remains higher than normal, the patient’s symptoms have resolved, and he no longer experiences itchiness, fatigue, dizziness, or syncope. He has returned to his normal daily activities with maintenance therapy of CsA (3 mg/kg/d). His renal function and hemoglobin levels are continuously monitored every 3 months. The timeline of this case is demonstrated in **Figure 2A**.

## Discussion

This brief report describes a PRCA patient with benign γδT cell proliferation and complex clonal TCR rearrangement in whom CsA corrected skin symptoms and hemoglobin levels. The main points of this interesting case are as follows. First, in this case, the γδT cell group associated with erythropoiesis suppression had unique morphological features and was captured for the first time under the microscope. In addition, this CD4+/CD8− (extremely high CD4/CD8 ratio) population is rare in PRCA patients. Second, our findings reveal the possibility that some PRCA patients may have clonal TCR rearrangement without a detectable malignant lymphoproliferative disease, and they are expected to respond to CsA with a good prognosis.

PRCA is a bone marrow failure with a marked reduction in erythroblasts caused by a T-lymphocyte-mediated autoimmune attack on erythropoietic stem cells (6). No existing diagnostic tests definitively establish PRCA, and diagnosis is currently made *via* systematic exclusion of various alternative etiologies. The reasoning and techniques throughout the diagnostic process of our case were instructive and provided insights into the future diagnosis of PRCA patients and the underlying cause of the disease.

Insufficient bone marrow erythropoiesis was highly suspected because of low reticulocyte levels and the exclusion of common causes of anemia. Bone marrow aspirate is the gold standard for PRCA diagnosis, while revealing the underlying cause of the disease is a process of exclusion. Human parvovirus B19, anti-erythropoietin antibodies, and thymoma were all excluded from investigations. Flow cytometry is a sensitive and specific technique that can identify any significant increase in a certain cell population and should be regarded as a norm for exploring PRCA causes. The significantly reduced level of this same group of γδT cells in the flow cytometry results after CsA treatment, together with the improved anemia and skin symptoms, confirmed the hypothesis that this group of proliferated γδT cells caused the patient’s PRCA.

Skin itchiness is a clinical sign that deserves more attention in practice, especially when observed in a PRCA patient or, more generally, a patient at risk of autoimmune or lymphoproliferative disorders. Skin itchiness is a potential sign of γδT cell proliferation in these patients. This assumption is further supported by skin irritation or inflammation commonly reported in cases of γδT cell proliferation or lymphoma; these cells reside in the skin tissue and play autoimmune roles when their homeostasis is disrupted (4). However, further studies are required to investigate whether skin itchiness could appear before other symptoms are noticed.

The γδT cells responsible for the patient’s disease were an atypical subtype. The most reported type of γδT cells in lymphoproliferative disorders that cause PRCA is large granular lymphocyte (LGL) (2), whose morphology is obviously distinct from the cells that we observed. Although large azurophilic cytoplasmic granules are observed in both cells, LGL, on the contrary, has more abundant basophilic cytoplasm (low nuclear/cytoplasmic ratio) with no protrusions. Regarding the immunophenotype, γδT cells are typically CD4−/CD8− or occasionally CD4−/CD8+, and this CD4+/CD8− (extremely high CD4/CD8 ratio) population was quite rare (7). However, the significance of this difference in morphology and immunophenotype and how these characteristics are associated with cell function has not been established. This γδT cell proliferation could not be categorized into any lymphoid disorder in the 4th edition of the World Health Organization (WHO) classification. However, in the latest 5th edition, autoimmune lymphoproliferative syndrome (ALPS) was proposed for the first time, and this new classification shares similar characteristics with our presented case (i.e., benign nature and lack of destructive infiltrate) (8).

There are several possible mechanisms by which this observed group of γδT cells is associated with erythropoiesis suppression. The most discussed theory is the disrupted balance between the proliferated innate immune cells and the physiological downregulation of HLA class I antigen on red-cell progenitors. The cells proliferated in our case were positive for CD94, one of the killer-cell inhibitory receptors (KIRs) that causes MHC-unrestricted cell death and only spares cells with adequate HLA class I antigen (2). Although we have not separately measured the levels of the two types of heterodimers, CD94/NKG2A and CD94/NKG2C, we expected to observe a higher ratio of the activating receptor NKG2C in this patient, disrupting the balance of cytotoxicity and destroying erythroid precursors. In other theories, red-cell destruction could be triggered when activated T cells recognize ligands on red-cell progenitors, although these ligands are not clearly described (2). As we highlighted above, the proliferative cells in our case were a rare CD4+CD8− subset. Although most CD4+ T-cell subsets are considered to have no cytotoxic effects, more recent studies have revealed that CD4 cytotoxic T lymphocytes (CD4 CTLs) are observed in various autoimmune diseases and secrete granzyme B and perforin, killing target cells in an MHC class II-restricted fashion (i.e., directly killing target cells in an antigen (Ag)-specific fashion upon direct contact) (9, 10). CD4 CTLs have also been observed to destroy the bone marrow in aplastic anemia patients (8, 10). In addition, these cells have a Th cytokine-producing role that can promote phagocytic activity, the generation of CD8 cytotoxic T cells, antibody production, and pro-inflammatory responses. Thus, the present case provides clinical evidence for this theory and challenges the restricted role of cytotoxicity to CD4−/CD8− or CD4−/CD8+ T cells. Cytotoxicity is also believed to be induced when antibodies crosslink the Fc receptor on innate immune cells with ligands on red-cell progenitors specific to that antibody. Interestingly, CD16 was also one of the Fc receptors found positive in our observed γδT cell group (11).

TCR clonal rearrangement is generally seen in malignant lymphoproliferative diseases but is also found in patients with Epstein–Barr virus infection, autoimmune diseases, immunodeficiency, and bone marrow transplantation (12). Rearrangements of the TCR-β and TCR-γ chains have been reported in PRCA associated with thymoma or T-cell large granular leukemia (13, 14). Others have reported oligoclonal expansion of T lymphocytes in PRCA patients not associated with LGL leukemia or thymoma (12). Thus, TCR gene rearrangement may play a role in the pathogenesis of PRCA. Moreover, patients presenting TCR-δ or/and TCR-γ gene rearrangement may respond poorly to CsA (5). Our patient’s TCR gene rearrangement demonstrated monoclonal patterns in several different TCR gene segments, indicating a monoclonal rearrangement in the polyclonal background, which is distinct from other reported clonal subtypes. In addition, although the patient had TCR clone changes, he responded well to CsA. It remains controversial whether the clonal expansion of γδT cells found in the present study can be generally found in acquired PRCA. The role of clonal TCR gene rearrangement in PRCA pathogenesis also remains unclear. Thus, further studies are needed to explore these aspects of PRCA.

## Conclusion

This rare case of PRCA in an elderly patient with benign proliferative γδT cells and clonal TCR rearrangement provides new insights into the diagnosis and management of PRCA in patients through flow cytometry and gene rearrangement in investigations. Furthermore, for PRCA cases in which a group of benign γδT cells is confirmed responsible, the number of these cells before and after treatment can provide a new basis for therapeutic efficacy and monitoring. However, more evidence on the specific mechanism by which this unique cell subset causes PRCA is needed.

## Data availability statement

The original contributions presented in the study are included in the article/Supplementary Material. Further inquiries can be directed to the corresponding author.

## Ethics statement

The studies involving human participants were reviewed and approved by The Medical Ethics Committee of The Second Affiliated Hospital, College of Medicine, Zhejiang University. The patients/participants provided their written informed consent to participate in this study. Written informed consent was obtained from the individual(s) for the publication of any potentially identifiable images or data included in this article.

## Author contributions

XL, XYZ, and WW contributed to the design and conception of the study. XL and XYZ contributed to data collection. XL contributed to writing the initial drafting of the manuscript. XHZ and WW reviewed and edited the original draft. All authors contributed to the manuscript revision and read and approved the submitted version.

## Supplementary material

Supplementary Figure 1 Flow cytometry results before treatment. The proportion of the abnormal γδT cell clone and other cell types in the patient’s bone marrow before treatment.

Supplementary Figure 2 Flow cytometry results after treatment. The proportion of the abnormal γδT cell clone and other cell types in the patient’s bone marrow after 10 months of treatment.

Supplementary Figure 3 Monoclonal rearrangement map of this patient (partial).Monoclonal TCR gene rearrangement was detected in TCRβ and TCRδ, while multiclonal patterns were also detected in TCRβ.
