# Supplementary figures and images for: Successful treatment of a pure red-cell aplasia patient with γδT cells and clonal TCR gene rearrangement: A case report
Source: Front Immunol. 2023 Jan 16;13:1103448. doi: 10.3389/fimmu.2022.1103448 (PMC9885080; doi:10.3389/fimmu.2022.1103448)

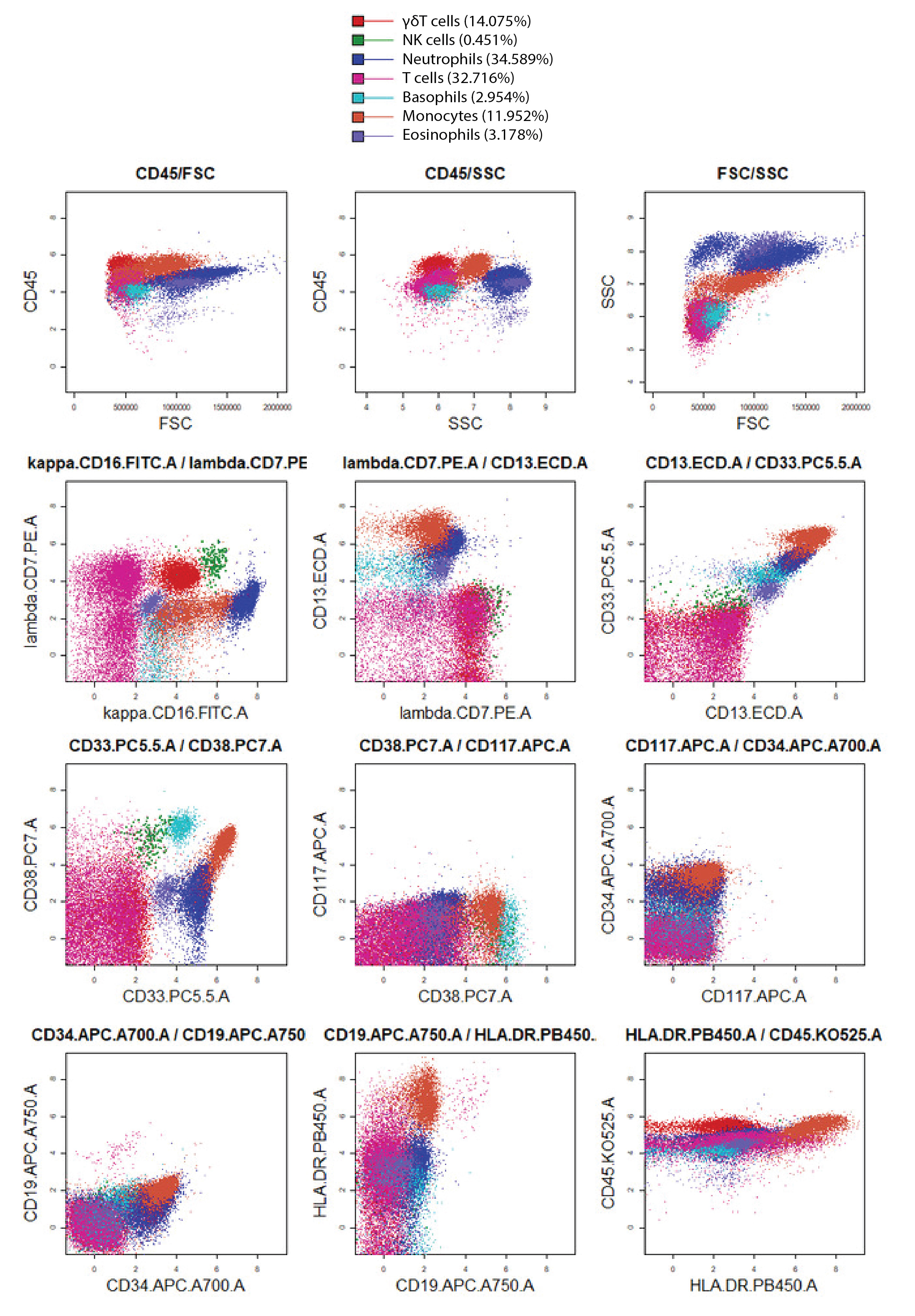

Supplement: Supplementary Figure 1 — Flow cytometry results before treatment. The proportion of the abnormal γδT cell clone and other cell types in the patient’s bone marrow before treatment. [file Image_1.jpeg]

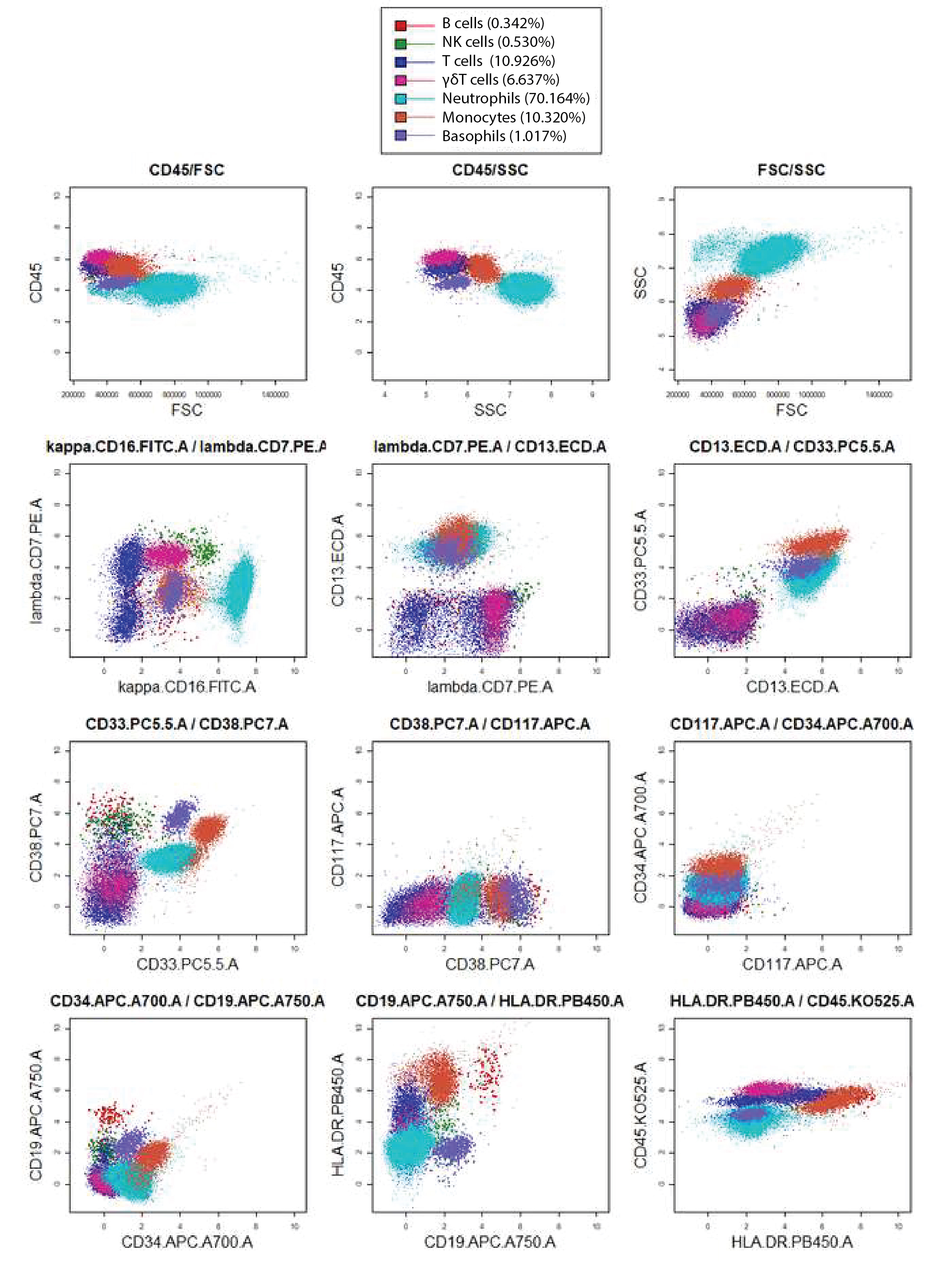

Supplement: Supplementary Figure 2 — Flow cytometry results after treatment. The proportion of the abnormal γδT cell clone and other cell types in the patient’s bone marrow after 10 months of treatment. [file Image_2.jpeg]

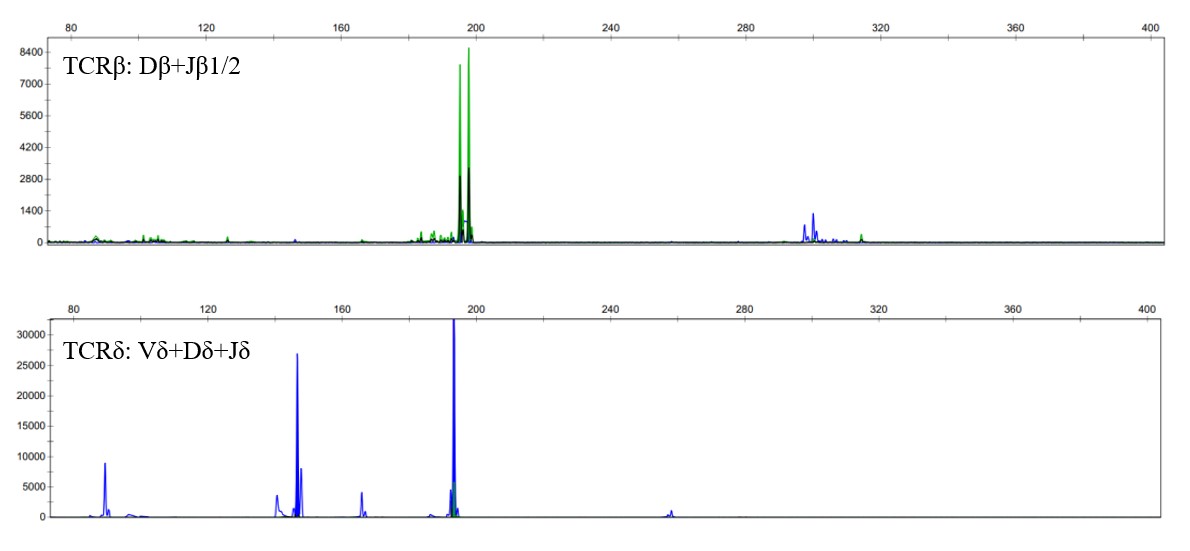

Supplement: Supplementary Figure 3 — Monoclonal rearrangement map of this patient (partial).Monoclonal TCR gene rearrangement was detected in TCRβ and TCRδ, while multiclonal patterns were also detected in TCRβ. [file Image_3.jpeg]
